# Supplementary material for: Comment on ‘Accumbens cholinergic interneurons dynamically promote dopamine release and enable motivation’
Source: bioRxiv. 2024 Jan 5:2023.12.27.573485. Preprint. [Version 2] doi: 10.1101/2023.12.27.573485 (PMC10802245; doi:10.1101/2023.12.27.573485)
Supplement: 1 [file NIHPP2023.12.27.573485V2-supplement-1.pdf]

bioRxiv preprint doi: https://doi.org/10.1101/2023.12.27.573485; this version posted January 5, 2024. The copyright holder for this preprint (which was not certified by peer review) is the author/funder, who has granted bioRxiv a license to display the preprint in perpetuity. It is made available under aCC-BY 4.0 International license.

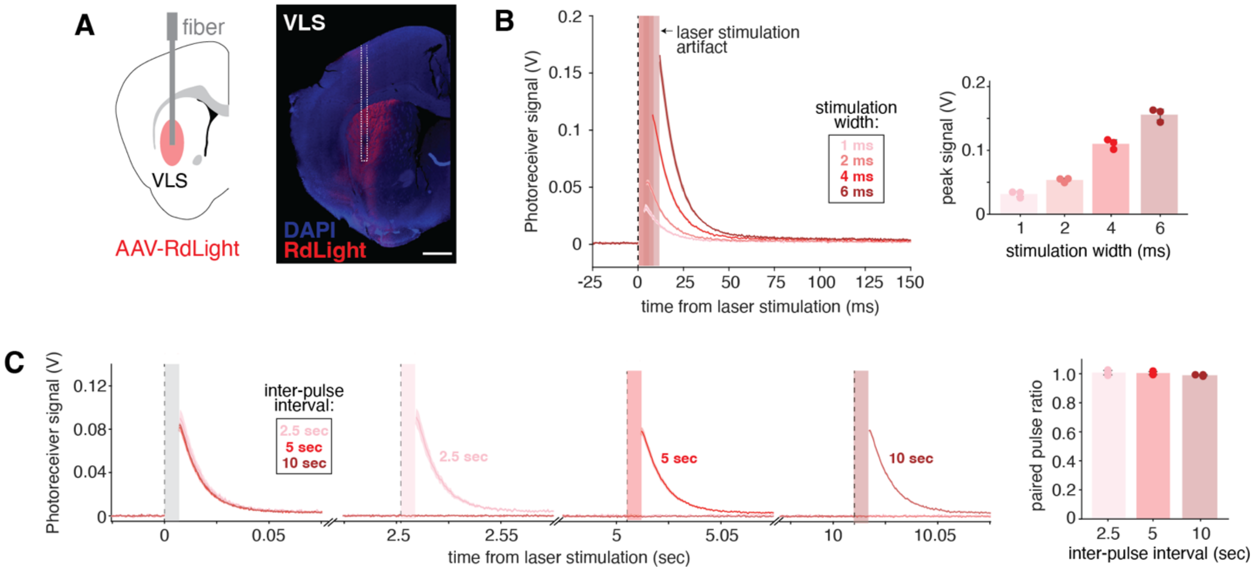

Figure 1 – Figure supplement 1: Blue light evokes RdLight1 photoactivation in VLS. (A) Injection and fiber implantation scheme for mice expressing RdLight1 in the VLS (left). Images of sensor expression from a representative mouse (right; scale bar: 800 µm). **(B)** Mean (± SEM) RdLight1 photoactivation signal in the VLS in response to 1, 2, 4, or 6 ms single pulses of blue light stimulation (10 mW) coupled with constant 565 nm illumination (30 µW). Colored vertical bars indicate laser stimulation artifacts which were removed. Mean maximal RdLight1 photoactivation signals recorded under the indicated stimulation widths are displayed. Each dot represents the average signal from a single mouse, and error bars denote the standard deviation (N = 3 mice). **(C)** Mean (± SEM) RdLight1 photoactivation signal in the VLS in response to a pair of 4 ms blue light pulses (10 mW) separated by the indicated inter-pulse intervals. Mean paired pulse ratios are displayed (magnitude of pulse #2/pulse #1), with each dot representing a single mouse, and the error bars denoting the standard deviation (N = 3 mice).
